# CUB domain-containing protein 1 signaling dysregulates gemcitabine metabolism contributing to therapeutic resistance in T24 cells

**DOI:** 10.1371/journal.pone.0331289

**Published:** 2025-09-02

**Authors:** Kun-Lin Hsieh, Kuan-Hua Huang, Ching-Ping Chang, Hung-Wen Tsai, Yu-Hao Chang, Yi-Ru Zheng, Huei-Sheng Huang

**Affiliations:** 1 Division of Urology, Department of Surgery, Chi Mei Medical Center, Tainan, Taiwan; 2 Department of Medical Research, Chi Mei Medical Center, Tainan, Taiwan; 3 Department of Pathology, National Cheng Kung University Hospital, College of Medicine, National Cheng Kung University, Tainan, Taiwan; 4 Department of Medical Laboratory Science and Biotechnology, College of Medicine, National Cheng Kung University, Tainan, Taiwan; Columbia University, UNITED STATES OF AMERICA

## Abstract

Gemcitabine is commonly used in the standard first-line treatment of urothelial carcinoma (UC); however, the emergence of drug resistance significantly limits its clinical benefit. The present study aims to investigate the role of CUB domain-containing protein 1 (CDCP1) in mediating resistance to gemcitabine in UC cells. Gemcitabine-resistant T24 (T24-GR) cells exhibited downregulation of human equilibrative nucleoside transporter 1 and upregulation of cytidine deaminase, key regulators of gemcitabine metabolism, as well as increased CDCP1 expression. Notably, silencing CDCP1 reversed these resistance-associated expression patterns. Mechanistically, T24-GR cells displayed elevated expression of CDCP1 and increased phosphorylation of c-Src and PKCδ, indicating activation of downstream survival signaling. Overexpression of CDCP1 in T24-CD cells activated similar pathways and modulated regulators of gemcitabine metabolism. In contrast, CRISPR/Cas9-mediated knockout of CDCP1 in T24-CDKO cells suppressed c-Src/PKCδ signaling and increased sensitivity to gemcitabine-induced cytotoxicity. Using flow cytometry, we observed that treatment with gemcitabine induced apoptosis in parental T24 cells, as indicated by an increase in the sub-G1 population. In contrast, T24-GR and T24-CD cells showed minimal sub-G1 accumulation, suggesting resistance to gemcitabine-induced apoptosis. Western blot analysis revealed decreased levels of cleaved caspase-3 and cleaved poly(ADP-ribose) polymerase in T24-GR and T24-CD cells following gemcitabine exposure, whereas these markers were upregulated in parental T24 and T24-CDKO cells. Furthermore, the knockdown of CDCP1 and the utilization of c-Src/PKCδ signaling inhibitors in T24-GR cells led to the restoration of sensitivity to gemcitabine. By suppressing apoptosis and altering drug metabolism pathways, highlighting CDCP1 as a potential therapeutic target for overcoming gemcitabine resistance in UC.

## Introduction

Urothelial carcinoma (UC) is the fourth estimated new cancer case and the eighth estimated death in men in the USA in 2024 [1]. UC grows from the inner surfaces of the urothelium of the urinary tract, renal pelvis, ureter, proximal urethra, and bladder, and mostly occurs in the urinary bladder (90–95%) [2]. The risk factors for UC include smoking, long-term exposure to arsenic water, aromatic amines, alkylation agents, and herbal medicines containing aristolochic acid [3]. UC can be classified into non-muscle-invasive UC (NMIUC) and muscle-invasive UC (MIUC). About 20–30% of patients with MIUC have higher rates of metastasis and mortality and need frequent diagnoses and treatments throughout their whole life [2]. Therefore, treating UC and its associated complications places a significant economic burden on the healthcare system [4].

So far, various strategies are available for UC therapy, including surgery, systemic chemotherapy, radiation therapy, and combination therapies. The standard first-line therapeutic agents include gemcitabine plus cisplatin (GC), or methotrexate/vinblastine/doxorubicin/cisplatin (MVAC) [5]. Especially, GC regimens express similar therapeutic effects but less substantial toxicities to MVAC regimens for locally advanced and metastatic UC (stage IV), strengthening its standard of care in patients with locally advanced or metastatic UC [6]. Gemcitabine is approved by the US Food and Drug Administration as a first-line therapy for patients with advanced pancreatic cancer, non-small cell lung cancer, breast cancer, and UC, due to its low toxicity profile, which is suitable for combination therapy, such as cisplatin and paclitaxel [7,8]. However, in clinical practice, about 50% of these patients express no response to the GC treatment, which causes the five-year survival rate to decrease to around 15% [6]. The chemoresistance and rapid relapse/recurrence are the major reasons for treatment failure of UC patients [5]. This reveals the need to understand GC-resistant mechanisms to develop a novel strategy for effective treatment.

Gemcitabine (2’, 2’-difluorodeoxycytidine, dFdC) is a deoxycytidine analog and antimetabolite chemotherapy agent. Gemcitabine is transported into cells by human equilibrative nucleoside transporters (hENTs) and then activated by deoxycytidine kinase (dCK) via phosphorylation to become dFdC-5´-monophosphate (dFdC-MP). Then the dFdC-MP is further metabolized to dFdC-5´-diphosphate (dFdC-DP), which inhibits ribonucleotide reductase M1 or M2 (RRM1/RRM2), leading to depletion of dCTP pools and increased phosphorylation of gemcitabine. The results facilitate the incorporation of dFdC-TP into DNA, inhibiting DNA synthesis and inducing cellular apoptosis [8]. It is reported that overexpression of RRM1 was associated with gemcitabine resistance in various cancers [8,9]. In contrast, gemcitabine is inactivated by cytidine deaminase (CDA) via conversion to difluorodeoxyuridine (dFdU) [10]. Evidence indicates that the silencing of hENTs and dCK can inhibit the entry of gemcitabine into cells and its subsequent activation, leading to resistance to the drug. In addition, increased CDA expression by m^6^A methyltransferase METTL14 causes gemcitabine resistance in pancreatic cancer [10,11].

CUB domain-containing protein 1 (CDCP1), also known as gp140, SIMA135, and TRASK, is a type I-transmembrane glycoprotein that acts as a receptor tyrosine kinase to relay oncogenic signals to cancer progression [12]. CDCP1 (135–140 kDa) comprises three domains: extracellular, transmembrane, and cytoplasmic. Cleavage of full-length CDCP1 at R368/K369 produces an amino-terminal fragment (ATF, 65 kDa) and a carboxyl-terminal transmembrane fragment (70 kDa) [13,14]. Five conserved tyrosine residues (Y707, Y734, Y743, Y762, and Y806), located at the cytoplasmic domain of CDCP1, can be phosphorylated by Src family kinase (SFK) to recruit PKCδ to form the p-CDCP1/SFK/PKCδ complex, which then regulates anoikis resistance, cellular migration/invasion, and ECM degradation [13,15,16]. CDCP1 has been proven to be a marker of hematopoietic stem cells with multilineage differentiation potential [17].

The widespread upregulation renders CDCP1 a significant clinical potential in cancers, serving as both a biomarker and a therapeutic target [12]. Overexpression of CDCP1 in various cancers, including cancer of the colon, lung, breast, renal, and pancreas, is highly correlated with cancer progression [12]; thus, it is expected to serve as a new biomarker for diagnosis. Elevated CDCP1 expression is frequently associated with poor prognosis, including tumor growth, metastasis, recurrence, and reduced overall survival in many cancers [18]. Increased CDCP1-ATF, resulting from proteolytic cleavage, has been detected in the serum of patients with colorectal and gastric cancer, and the urine of prostate cancer patients [18]. We also demonstrated that CDCP1 promotes the malignant progression of UC and has the potential to be used as a urine-based biomarker for the diagnosis of low-grade UC [19].

CDCP1 is a promising therapeutic target due to its widespread overexpression in tumors and restricted expression in numerous normal tissues. Antibody-based therapies targeting CDCP1 are currently being investigated in preclinical settings, demonstrating promise in inhibiting tumor growth and metastasis. This molecule is also being explored as a target for theranostic applications, which integrate diagnostic imaging (e.g., PET-CT) with radiolabeled antibody-based targeted therapy [20]. In addition, increased expression of CDCP1 is associated with drug resistance in several *in vitro* and preclinical modeling studies *in vivo* [18]. However, the mechanisms of CDCP1 in chemoresistance of UC must be well-elucidated.

In the current study, we generated gemcitabine-resistant, CDCP1-overexpressed, and CDCP1-silenced UC cells to investigate the role of CDCP1 expression in therapeutic resistance. We demonstrated that CDCP1 modifies the expression of metabolic genes related to gemcitabine through the c-Src/PKCδ signaling pathway, thereby reducing sensitivity to the drug.

## Materials and methods

### Reagents and antibodies

RPMI 1640 medium, fetal bovine serum (FBS), antibiotic-antimycotic (100X), trypsin-EDTA (0.5%), puromycin, and blasticidin were obtained from Gibco (Grand Island, NY, USA). JetPRIME® transfection reagent was sourced from Polyplus (Strasbourg, France). RNAzol® RT was procured from the Molecular Research Center (Cincinnati, OH, USA). The MMLV reverse transcription kit was purchased from Promega (Madison, WI, USA). Power SYBR™ Green PCR Master Mix was acquired from Applied Biosystems (Foster City, CA, USA). Halt™ Protease and Phosphatase Inhibitor Cocktail was obtained from Thermo Fisher Scientific (Cleveland, OH, USA). Immobilon-P PVDF membrane and enhanced chemiluminescence reagent were sourced from Millipore (Billerica, MA, USA). Primary antibodies against pCDCP1^Tyr734^ (cat#9050, 1:1000), CDCP1 (cat#4115, 1:1000), c-Src (cat#2108, 1:1000), pSrc^Tyr416^ (cat#2101, 1:1000), PKCδ (cat#2058, 1:1000), pPKCδ^Tyr311^ (cat#2055, 1:1000), caspase-3 (cat#9662, 1:1000), and cleaved PARP (cat#9541, 1:1000) were sourced from Cell Signaling Technology (Danvers, MA, USA). The antibody against CDA (ab222515, 1:1000) was purchased from Abcam (Cambridge, UK). Antibodies against hENT1 (SAB5500117, 1:1000), *β*-actin (A2228, 1:10000), and Flag (F3165, 1:10000) were obtained from Sigma-Aldrich (St. Louis, MO, USA). The primary antibody against α-tubulin (11224–1-AP, 1:3000) was sourced from Proteintech (Chicago, IL, USA). 3-(4,5-Dimethylthiazol-2-yl)-2,5-diphenyltetrazolium bromide (MTT) was obtained from Cyrusbioscience (Taipei, Taiwan).

The specific inhibitors for c-Src (Src inhibitor 1) and PKCδ (rottlerin) were obtained from Sigma-Aldrich (St. Louis, MO, USA).

### Cell culture

The T24 human bladder cancer cell line and HEK-293T cells were obtained from the Bioresource Collection and Research Center (Hsinchu, Taiwan). T24-GR cells were established as described in a previous study [21]. These cells were cultured in RPMI 1640 medium supplemented with 10% FBS and an antibiotic-antimycotic solution and maintained in a humidified atmosphere with 5% CO_2_ at 37°C.

### Quantitative real-time PCR (qPCR)

Total RNA was isolated from T24 cells using RNAzol® and reverse-transcribed into cDNA following the manufacturer’s instructions for the MMLV reverse transcription kit. Specific primers for the targeted genes were designed and used for qPCR analysis, as described below: *cdcp1*-F: 5’-CAGGTGAAGCAGAACATCTCGG-3’, *cdcp1*-R: 5’-GTCACCGTGAAAACGCCTTCC-3’, *cda*-F: 5’-GGGTACAAGGATTTCAGGGCA-3’, *cda*-R: 5’-GTCATGTACACGGGCCAGTTG-3’, *hent1*-F: 5’-CAGGCAAAGAGGAATCTGGAG-3’, *hent1*-R: 5’-CAACAGTCACGGCTGGAAACA-3’, *β-actin*-F: 5’-TCCCTGGAGAAGAGCTACGA-3’, and *β-actin*-R: 5’-ACTCCATGCCCAGGAAGG-3’. The qPCR reactions were performed using Power SYBR™ Green PCR Master Mix on an Applied Biosystems platform. The relative expression levels of target genes were normalized to the internal control gene and calculated using the 2^−ΔΔCt^ method.

### Western blot analysis

Cells were lysed using a lysis buffer prepared by combining RIPA buffer with Halt™ Protease and Phosphatase Inhibitor Cocktail to prevent protein degradation and phosphorylation. The RIPA buffer consisted of 50 mM Tris-HCl (pH 7.5), 150 mM NaCl, 1% IGEPAL CA-630, and 0.5% sodium dodecyl sulfate (SDS), ensuring effective cell lysis and protein solubilization. Forty μg of extracted protein was separated by 10% SDS-polyacrylamide gel electrophoresis (SDS-PAGE). The resolved proteins were transferred onto Immobilon-P PVDF membranes for subsequent detection and analysis. The membranes were blocked with a 5% skim milk solution in TBST and incubated at room temperature for 1 hour. After blocking, the membranes were washed with TBST and incubated with primary antibodies overnight at 4°C. The following day, the membranes were probed with HRP-conjugated rabbit or mouse secondary antibodies. Immunoblots were visualized using an enhanced chemiluminescence reagent and imaged with an LAS-4000 imager (Fujifilm, Tokyo, Japan). The expression intensity of specific proteins was quantified using ImageJ software, with *β*-actin or *α*-tubulin expression as an internal control for normalization.

### Plasmid construction and lentivirus transduction

The pLenti-CDCP1 plasmid was constructed following the methodology described in a previous study [19]. Lentiviral particles were produced following standard protocols. Specifically, HEK-293T cells were transfected with the plasmids pCMV-8.91, pMD.G, and pLenti-CDCP1 using the jet PRIME transfection reagent, adhering to the manufacturer’s instructions. After an 18-hour transfection period, the medium was replaced with DMEM supplemented with 20% FBS for an additional 18 hours. The lentiviral supernatant was then harvested for subsequent transduction. T24 cells were transduced with the lentiviral medium containing 8 μg/ml polybrene. After an 18-hour transduction period, T24 cells were selected using 10 μg/ml blasticidin for 14 days. In addition, specific shRNA plasmids targeting the CDCP1 gene were obtained from the National RNAi Core Facility located at the Institute of Molecular Biology/Genomic Research Center, Academia Sinica (Taipei, Taiwan). The manipulation was carried out according to previous descriptions [19].

### Transient transfection

For the transfection assay, plasmids were combined with Lipofectamine™ 2000 reagent in 1 mL of Opti-MEM and incubated at room temperature for 30 minutes. The cells were then exposed to this mixture and incubated at 37°C in a humidified air/CO_2_ atmosphere (19:1) for 48 hours. Following this incubation period, the cells were lysed to determine protein expression using Western blot analysis.

### Establishment of CDCP1-knockout T24 cells

The CRISPR/Cas9 system was utilized to disrupt CDCP1 expression in T24 cells. A guide RNA targeting the exon of CDCP1 (5’-CGATAGAGACCCCGCAGTTC-3’) was cloned into the pAll-Cas9.Puro vector (Academia Sinica-RNAi Core Facility, Taipei, Taiwan) and transfected into T24 cells using a transfection reagent for 18 hours. Following transfection, the cells were treated with 10 μg/mL puromycin to select stable transfectants for 14 days. The CDCP1-knockout cells were further enriched through serial dilution and single-cell culturing. The knockout efficiency was subsequently validated using Western blot analysis. This approach successfully generated a stable CDCP1-knockout T24 cell line for subsequent functional analyses.

### Cell viability assay

Cell viability of T24 cells was assessed using the MTT colorimetric assay. Briefly, 1x10^5^ T24 cells were seeded in a 96-well plate with 10% FBS RPMI 1640 medium. After 24 hours, the medium was replaced with a 10% FBS RPMI 1640 medium containing gemcitabine, and the cells were incubated for an additional 48 hours. Following incubation, the medium was replaced with 1 mg/mL MTT in RPMI for 2 hours. The supernatant was carefully removed, and the MTT formazan was dissolved in DMSO. The absorbance was measured at 570 nm using a BioTek Epoch spectrophotometer (Winooski, VT, USA).

### Flow cytometry

Cells were seeded in 6 cm dishes and treated with 1000 nM gemcitabine. Cells in the logarithmic growth phase were harvested by trypsinization and washed with PBS. The collected cells were fixed in 75% ethanol at 4°C overnight. Following fixation, the cells were washed with PBS and incubated with DNase-free RNase A for 30 minutes at 37°C. Subsequently, the cells were stained with propidium iodide (PI) solution for 30 minutes at room temperature in the dark. Flow cytometric analysis was conducted using a CytoFLEX system (Beckman Coulter, Brea, USA), and 10000 cells were recorded per sample.

### Statistical analysis

All experiments were repeated at least three times. The statistical analysis was performed using Student’s t-test. All values are displayed as means ± SD for three determinations, with statistical significance indicated as follows: *p < 0.05, **p < 0.01, ***p < 0.001

## Results

### Gemcitabine-resistant T24-GR cells were generated

**Fig 1:**
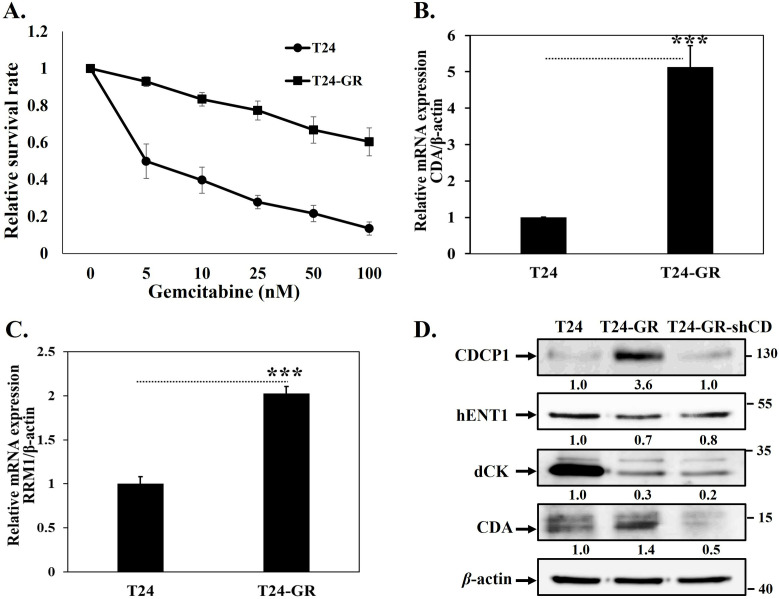
Gemcitabine-resistant T24-GR cells exhibit metabolic genes and CDCP1 expression. We treated T24 and T24-GR cells with gemcitabine for 48 hours to assess cellular viability using the MTT assay. The relative survival rates of (A) T24 and T24-GR cells are presented. qPCR analysis showing elevated mRNA expression levels of (B) *CDA* and (C) *RRM1* in gemcitabine-resistant T24-GR cells compared to parental T24 cells. (D) Western blot analysis of hENT1, dCK, and CDA protein expression in T24 and T24-GR cells. *β*-actin was used as a loading control. Data are presented as mean ± SD with statistical significance indicated as follows: *p < 0.05, **p < 0.01, ***p < 0.001.

Gemcitabine-resistant UC cells were established from the parental T24 cells by exposure to stepwise increasing concentrations of gemcitabine for up to 6 months. The concentration of gemcitabine used for treating T24 cells ranged from 10 nM to 1000 nM. Then we treated T24 and T24-GR cells with various concentrations of gemcitabine for 48 hours to measure cellular viability using the MTT assay. The results indicate that the survival rate of T24-GR is higher than that of the parental cells (Fig 1A). An increase in CDA and RRM1, along with a decrease in hENTs and dCK expression, has been shown to contribute to gemcitabine resistance [8–11]. Therefore, we measured the expression of CDA and RRM1 in both cell lines, which revealed elevated mRNA expression of CDA and RRM1 (Figs 1B-1C), along with downregulated protein expression of hENT1 and dCK in the T24-GR cells (Fig 1D). The upregulation of CDA was reversed in T24-GR cells after treatment with specific shRNA of CDCP1 (Fig 1D). These findings imply that CDCP1 might be involved in the gemcitabine resistance of UC cells via its regulation of CDA.

### CDCP1 and its downstream signaling are highly expressed in T24-GR cells

**Fig 2:**
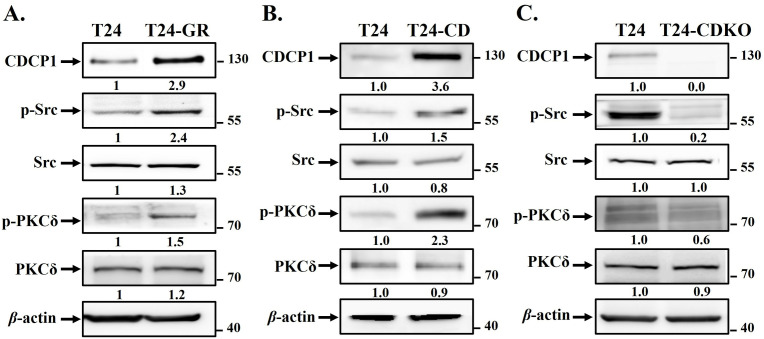
CDCP1/c-Src/PKCδ signaling pathway was upregulated in T24-GR cells. (A) Western blot analysis showing elevated protein expression of CDCP1, phospho-Src^Tyr416^, and phospho-PKCδ^Tyr311^ in T24-GR cells compared to parental T24 cells. Western blot analysis of CDCP1 and its downstream signaling components in T24, T24-CD, and T24-CDKO cells. (B) Overexpression of CDCP1 in T24-CD cells resulted in increased activation of c-Src and PKCδ, while (C) CDCP1 knockout in T24-CDKO cells reduced phosphorylation of both signaling proteins. These results suggest that CDCP1 modulates gemcitabine resistance through the c-Src/PKCδ pathway. *β*-actin was used as an internal control.

It has been reported that CDCP1 regulates tumor progression during metastasis through c-Src/PKCδ pathway and FAK/PI3K/AKT signaling [13,15,16,22]. Therefore, we evaluated the correlation between CDCP1 downstream signaling and gemcitabine resistance in UC cells. Using Western blot, the expression levels of CDCP1 protein and its downstream signaling, phosphor-Src and phosphor-PKCδ, in T24-GR cells were higher than in parental T24 cells (Fig 2A). Furthermore, we constructed the pLenti-CDCP1 plasmid and infected T24 cells using lentivirus transduction to obtain CDCP1-overexpressed T24 cells (T24-CD). We silenced CDCP1 in T24 cells using the CRISPR/Cas9 to obtain CDCP1-silenced T24 cells (T24-CDKO). The results demonstrated that CDCP1 regulates the c-Src/PKCδ signaling pathway in T24 cells (Figs 2B-2C), which is consistent with the findings in BFTC905 UC cells [19]. This indicates that the CDCP1/c-Src/PKCδ complex can be stimulated by gemcitabine in UC cells.

### CDCP1 regulates CDA and hENT1 expression through c-Src/PKCδ signaling

**Fig 3:**
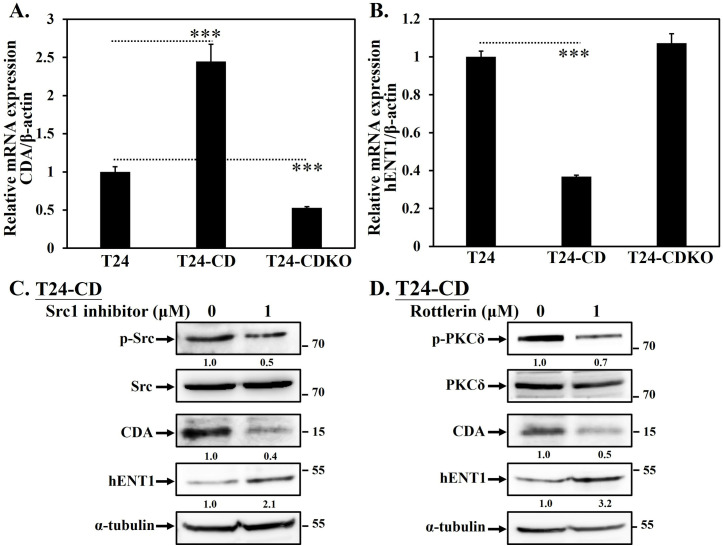
CDCP1 regulates CDA and hENT1 expression through c-Src/PKCδ signaling pathway. (A, B) Quantitative PCR analysis of CDA and hENT1 mRNA expression levels in T24, T24-CD, and T24-CDKO cells. T24-CD cells exhibited increased CDA and decreased hENT1 expression, whereas T24-CDKO cells showed the opposite trend. (C, D) Western blot analysis of CDA and hENT1 protein expression in T24-CD cells treated with a Src1 inhibitor or Rottlerin, respectively. phosphor-Src inhibition reduced CDA expression, while phosphor-PKCδ inhibition restored hENT1 expression. These findings suggest that CDCP1 modulates gemcitabine metabolism through activation of the c-Src and PKCδ signaling pathway. Data are presented as mean ± SD with statistical significance indicated as follows: *p < 0.05, **p < 0.01, ***p < 0.001.

To further investigate the role of CDCP1 in gemcitabine resistance, we analyzed the expression of CDA and hENT1 in T24, T24-CD, and T24-CDKO cells. Western blot analysis revealed that CDA expression was markedly increased in T24-CD cells, while significantly reduced in T24-CDKO cells (Fig 3A). Conversely, hENT1 expression was downregulated in T24-CD cells but modestly upregulated in T24-CDKO cells (Fig 3B). To assess the involvement of CDCP1 downstream signaling, we treated T24-CD cells with a Src inhibitor (Src inhibitor 1) and a PKCδ inhibitor (rottlerin). Inhibition of Src activity attenuated the upregulation of CDA (Fig 3C), whereas inhibition of PKCδ restored hENT1 expression (Fig 3D). These findings indicate that CDCP1 upregulates CDA and downregulates hENT1 through activation of the c-Src/PKCδ signaling pathway.

### Overexpression of CDCP1 prevents gemcitabine-induced cellular apoptosis

**Fig 4:**
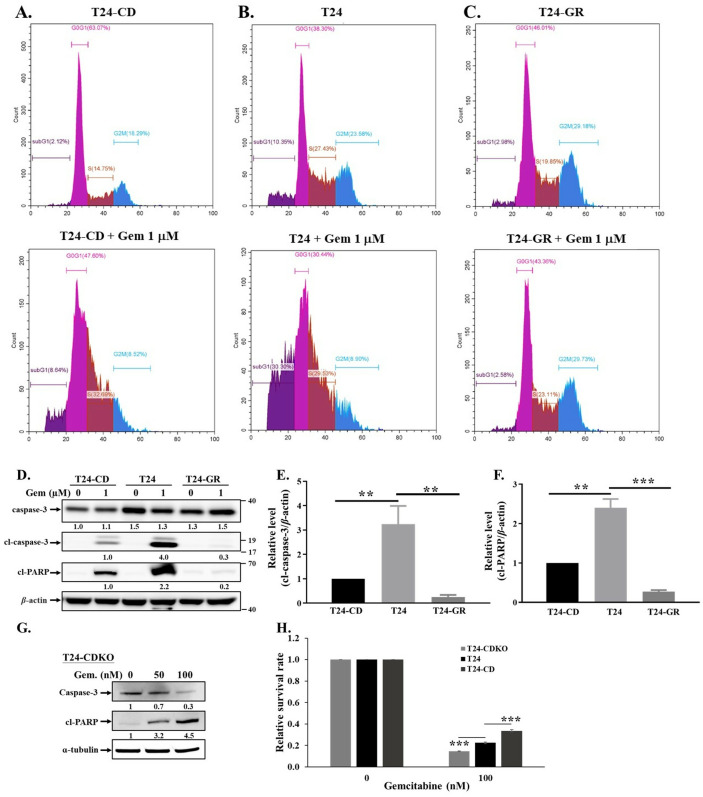
CDCP1 expression diminishes gemcitabine-induced apoptosis. To investigate the role of CDCP1 in gemcitabine-induced apoptosis in UC cells, we performed sub-G1 population analysis by flow cytometry with PI staining. (A) T24-CD, (B) T24, and (C) T24-GR cells were treated with 1 μM gemcitabine for 48 hours, followed by PI staining and cell cycle analysis. The percentage of sub-G1 population was quantified to evaluate DNA fragmentation. In addition, (D) cells were treated with 1 μM gemcitabine for 48 hours, and whole cell lysates were subjected to Western blot for analyzing caspase-3, cl-caspase-3, and cl-PARP expression. The *β*-actin was used as a loading control. (E, F) Quantification of cl-caspase-3 and cl-PARP protein levels from three independent experiments was shown. (G) In addition, T24-CDKO cells were treated with increasing concentrations of gemcitabine (0, 50, and 100 nM) for 48 hours. Cell lysates were analyzed for expression levels of caspase-3 and cl-PARP. Alpha-tubulin was used as a loading control. (H) T24, T24-CD, and T24-CDKO cells were treated with 0 or 100 nM gemcitabine for 72 hours using a cell viability assay. Viability was assessed using the MTT assay, as described in the “Materials and Methods.” Data represent mean ± SD from three independent experiments. P < 0.05 was considered statistically significant. Data are presented as mean ± SD with statistical significance indicated as follows: *p < 0.05, **p < 0.01, ***p < 0.001.

Cells were treated with gemcitabine for 48 hours, followed by PI staining and cell cycle analysis using flow cytometry. Apoptosis was evaluated by quantifying the sub-G1 population, which reflects DNA fragmentation, a hallmark of apoptotic cell death [23]. T24-GR cells exhibited resistance to gemcitabine-induced apoptosis, as evidenced by the absence of a significant increase in the sub-G1 fraction following treatment (Fig 4C). Similarly, T24-CD showed no significant elevation in sub-G1 population compared to parental T24 cells (Figs 4A-4B), indicating a potential role of CDCP1 in attenuating gemcitabine-induced apoptotic responses.

In addition, caspase-mediated apoptosis has been implicated in the antitumor effects of gemcitabine in UC cells [24,25]. To investigate the involvement of CDCP1 in gemcitabine-induced apoptosis, T24, T24-GR, and T24-CD cells were treated with gemcitabine for 48 hours, followed by Western blot analysis of caspase-3 and cleaved poly(ADP-ribose) polymerase (cl-PARP), two well-established markers of apoptosis [26]. In T24 cells, gemcitabine treatment led to decreased pro-caspase-3 levels and increased cleaved-caspase-3 and cl-PARP expression, indicating activation of the apoptotic pathway (Fig 4D, lane 4). In contrast, T24-CD and T24-GR cells exhibited substantial reductions in cl-caspase-3 and cl-PARP levels after treatment (Fig 4D, lanes 2 and 6), suggesting enhanced resistance to apoptosis. We performed three independent experiments and quantified the results for statistical analysis (Figs 4E-4F). Furthermore, dose-dependent increases in cl-PARP and decreases in caspase-3 in T24-CDKO were observed, indicating restored apoptotic sensitivity following CDCP1 deletion (Fig 4G). The survival rates among these cells were also measured by using an MTT assay. T24-CDKO cells showed significantly reduced viability compared to T24 and T24-CD cells under gemcitabine treatment, supporting a role for CDCP1 in promoting chemoresistance (Fig 4H).

### Downregulation of the CDCP1/c-Src/PKCδ signaling pathway restores the sensitivity of resistant cells to gemcitabine

**Fig 5:**
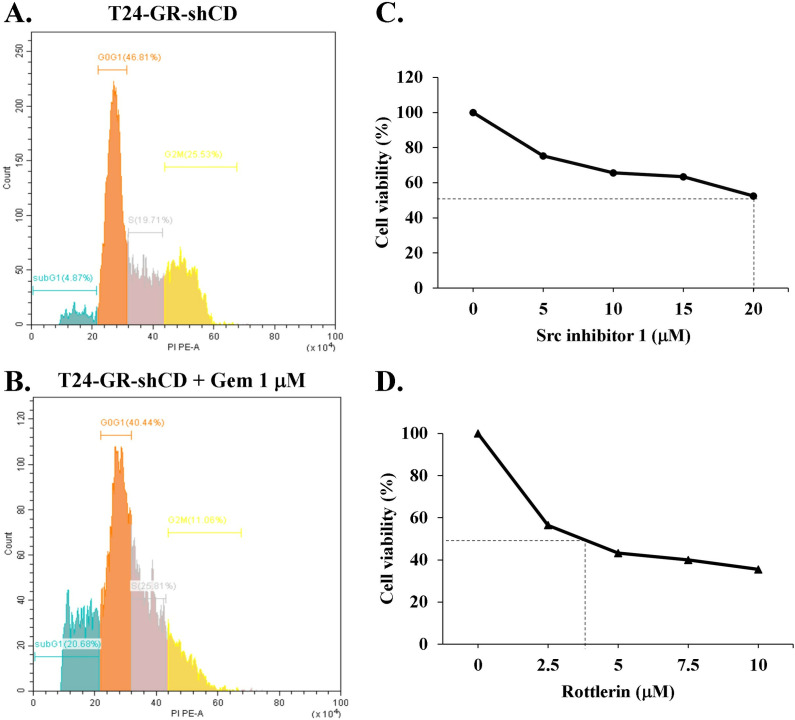
Suppression of the CDCP1/c-Src/PKCδ signaling pathway restores gemcitabine sensitivity in T24-GR cells. Flow cytometry analysis of the sub-G1 population was performed in T24-GR-shCD cells treated with (A) vehicle or (B) 1 μM gemcitabine for 48 hours. Quantification of sub-G1 populations showing a significant increase from 4.86% to 20.68% upon CDCP1 knockdown and gemcitabine treatment (4.25-fold increase). T24-GR cells were treated with increasing concentrations of the (C) Src inhibitor 1 or (D) rottlerin (a PKCδ inhibitor) for 48 hours, and viability was assessed by MTT assay. Both inhibitors significantly reduced cell survival in a dose-dependent manner.

To investigate whether suppression of CDCP1 could restore gemcitabine sensitivity in resistant cells, we knocked down CDCP1 in T24-GR cells (T24-GR-shCD) and assessed their response to gemcitabine. Flow cytometry analysis revealed a substantial increase in the sub-G1 population from 4.86% to 20.68%, representing a 4.25-fold elevation (Figs 5A-5B). CDCP1 knockdown markedly increased the sub-G1 fraction, indicating enhanced apoptosis. In contrast, T24-GR cells without CDCP1 knockdown exhibited minimal changes in the sub-G1 population upon gemcitabine treatment (2.98% to 2.58%), indicating persistent resistance (Fig 4C). Additionally, treatment of T24-GR cells with either Src inhibitor 1 or rottlerin resulted in a dose-dependent reduction in cell viability (Figs 5C-5D). Collectively, these results suggest that inhibition of the CDCP1/c-Src/PKCδ signaling pathway effectively enhances the responsiveness of T24-GR cells to gemcitabine.

## Discussion

In the era of precision medicine, various novel therapeutic strategies are available for UC treatment, including immune checkpoint inhibitors (PD1/L1 inhibitors), antibody-drug conjugates, and targeted therapy. Unfortunately, their modest outcomes in cisplatin-ineligible patients need further investigation [27]. Gemcitabine is an antimetabolite that has been approved for cancer chemotherapy since 1996. Antimetabolites represent one of the most effective classes of anticancer drugs, specifically targeting RNA and DNA. However, drug resistance frequently develops, highlighting the need for the design of new antimetabolites or the exploration of combination therapies with other anticancer agents. [28].

The mechanism of gemcitabine resistance is explored in some cancer cells, such as pancreatic cancer [10,11], lung cancer [9], breast cancer [29], cholangiocarcinoma [30], and UC [21]. Various degrees of chemoresistance have been observed after treatment with gemcitabine-based agents in UC patients, which limits the efficacy of the therapy [31]. Therefore, it is crucial to elucidate the mechanisms underlying gemcitabine resistance to develop effective treatment strategies for UC patients. A deeper understanding of gemcitabine resistance mechanisms would enable us to optimize therapeutic strategies for UC.

In the present study, we found that T24-GR cells expressed a higher protein level of CDCP1 than wild-type T24 cells (Fig 2A). Furthermore, T24-CD cells exhibited greater resistance to gemcitabine. In contrast, T24-CDKO cells displayed lower gemcitabine resistance than wild-type T24 cells (Fig 4). Specific silencing of CDCP1 with shRNA in T24-GR cells re-sensitized them to gemcitabine (Figs 5A-5B). These results indicate a positive correlation between CDCP1 expression and gemcitabine resistance in UC. CDCP1 is a critical mediator of ovarian clear cell carcinoma progression, promoting spheroid formation, migration, and chemoresistance. Therapeutic targeting of CDCP1 may improve outcomes for patients with this aggressive form of ovarian cancer [32]. It also interacts with the loss of the tumor suppressor gene PTEN to facilitate the development of castration-resistant prostate cancer [33]. CDCP1 disrupts lipid metabolism by reducing the abundance of lipid droplets and promoting fatty acid oxidation, which contributes to the migration and metastasis of triple-negative breast cancer [34]. Our previous results revealed that CDCP1 induces malignant progression in UC and may serve as a urine-based biomarker for detecting both low- and high-grade UC [19]. The current findings indicate that UC patients with high CDCP1 expression have a poor prognosis, underscoring the need to optimize therapeutic strategies to enhance treatment efficacy.

In addition, CDCP1 can alter gemcitabine metabolism via the c-Src/PKCδ pathway (Figs 3C-3D), contributing to resistance (Figs 5C-5D). As described above, gemcitabine undergoes a complex metabolic process involving several genes. The silencing of hENTs and dCK inhibits gemcitabine uptake and activation, preventing it from entering cells and being phosphorylated into its active state. This leads to decreased intracellular levels of active gemcitabine, resulting in resistance [8]. Overexpression of genes involved in gemcitabine inactivation, such as CDA and 5’-NT, promotes the conversion of gemcitabine into inactive metabolites, reducing its efficacy [10,11]. Enhanced DNA repair mechanisms, mediated by genes like RRM1/RRM2, mitigate the cytotoxic effects of gemcitabine-induced DNA damage [8,9]. Various levels of CDCP1 expression in T24 cells regulate hENT1 and CDA expression (Figs 1 and 3), mediated by the c-Src/PKCδ signaling pathway (Fig 3). Higher hENT1 levels facilitate greater drug uptake, while lower CDA activity corresponds to less drug inactivation. Analyzing these changes is important because it indicates that targeting CDCP1 can reverse chemoresistance in UC cells by improving gemcitabine uptake and reducing its inactivation, potentially offering a therapeutic strategy for gemcitabine-resistant patients. In addition to our findings in T24 cells, we have also shown that the CDCP1/c-Src/PKCδ pathway is critical to the progression of BFTC905 UC cells [19]. Targeting specific genes or pathways involved in gemcitabine resistance may help sensitize cancer cells to treatment and improve patient outcomes.

Furthermore, we demonstrated that the activated AKT/GSK3β pathway contributes to the gemcitabine resistance in UC cells through the hedgehog (HH) pathway [21]. By proteases, CDCP1 overexpression and proteolytic cleavage can initiate FAK/PI3K/AKT signaling, promoting metastasis in cellular and animal models [13,22]. Several stemness markers have been associated with cancer stemness and tumorigenesis in UC, including Oct4, CD133, SOX4, ALDH1A1, and components of the HH signaling pathway. We speculate that CDCP1 may regulate stemness in UC through the AKT/HH pathway, contributing to therapeutic resistance. The regulation of stemness by CDCP1 in UC remains an area for future investigation.

**Fig 6:**
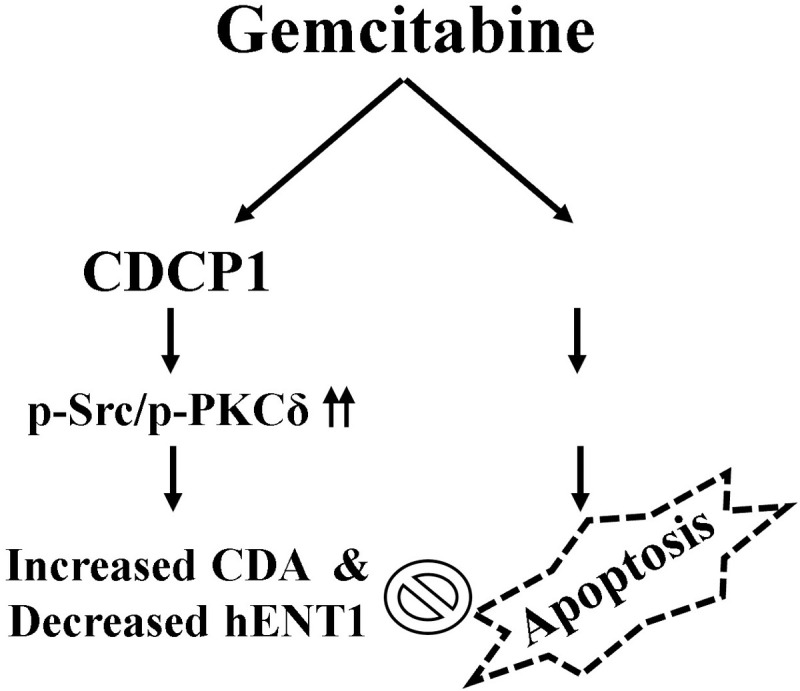
Schematic summary of CDCP1-mediated gemcitabine resistance in UC cells. This model illustrates the role of CDCP1 in regulating gemcitabine sensitivity through modulation of drug metabolism and apoptotic signaling. In gemcitabine-sensitive T24 cells, treatment leads to efficient drug uptake via CDA and hENT1, resulting in increased apoptosis characterized by caspase-3 activation, PARP cleavage, and accumulation of cells in the sub-G1 phase. In contrast, gemcitabine-resistant T24-GR and CDCP1-overexpressing T24-CD cells exhibit elevated CDCP1 expression, which activates downstream c-Src and PKCδ signaling, upregulates CDA and downregulates hENT1 expression, and suppresses apoptotic markers, contributing to drug resistance. Knockout of CDCP1 in T24-CDKO cells reverses this phenotype, restoring gemcitabine sensitivity via reactivation of apoptosis and decrease of CDA and re-expression of hENT1. These findings highlight CDCP1 as a critical modulator of gemcitabine resistance through both metabolic and apoptotic pathways.

Gemcitabine can enhance the efficacy of immunotherapies against cancers that are traditionally resistant to treatment through several key mechanisms, including inducing immunogenic tumor cell death, promoting antigen presentation, reducing immunosuppressive cells, and sensitizing tumors to T cell killing [28]. However, optimizing the sequencing and scheduling of gemcitabine with immunotherapies is key to harnessing these synergistic effects. As described, CDCP1 plays essential roles in promoting cancer cell survival, growth, metastasis, and resistance to chemotherapy and targeted agents [18]. Our findings link CDCP1 to gemcitabine resistance in UC cells (Fig 6). Understanding the role of CDCP1 in regulating therapeutic resistance to gemcitabine could have significant clinical implications for UC management. Targeting the CDCP1 signaling pathway, potentially in combination with conventional therapies or immunotherapies, may help overcome treatment resistance and improve outcomes for patients with aggressive, CDCP1-positive UC. Nevertheless, preclinical data are limited and encouraging across various cancers. More preclinical experiments and further clinical validation are needed to determine optimal approaches, efficacy, and safety for clinical practice.

## Supporting information

S1 Fig Gemcitabine-resistant T24-GR cells exhibit metabolic genes and CDCP1 expression. (PDF)

S2 Fig CDCP1/c-Src/PKCδ signaling pathway was upregulated in T24-GR cells. (PDF)

S3 Fig CDCP1 regulates CDA and hENT1 expression through c-Src/PKCδ signaling pathway. (PDF)

S4 Fig CDCP1 expression diminishes gemcitabine-induced apoptosis. (PDF)
