# Supplementary figures and images for: CUB domain-containing protein 1 signaling dysregulates gemcitabine metabolism contributing to therapeutic resistance in T24 cells
Source: PLoS One. 2025 Sep 2;20(9):e0331289. doi: 10.1371/journal.pone.0331289 (PMC12404478; doi:10.1371/journal.pone.0331289)

Fig 1

A.

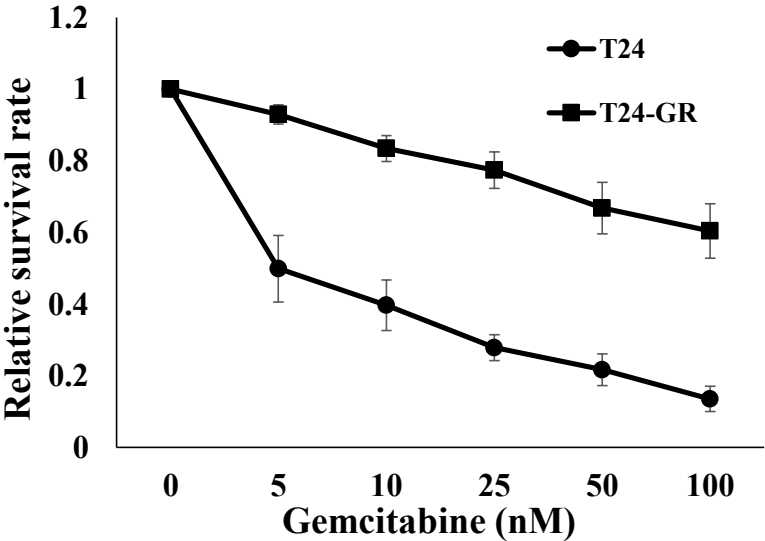

|     |   |          |          |          |          |          |
|-----|---|----------|----------|----------|----------|----------|
| T24 |   |          |          |          |          |          |
|     | 1 | 0.492537 | 0.358209 | 0.253731 | 0.19403  | 0.134328 |
|     | 1 | 0.507937 | 0.428571 | 0.333333 | 0.269841 | 0.174603 |
|     | 1 | 0.633333 | 0.416667 | 0.333333 | 0.316667 | 0.2      |
|     | 1 | 0.379518 | 0.305221 | 0.248996 | 0.198795 | 0.13253  |
|     | 1 | 0.384458 | 0.312883 | 0.271984 | 0.210634 | 0.139059 |
|     | 1 | 0.389452 | 0.3286   | 0.249493 | 0.212982 | 0.129817 |
|     | 1 | 0.591549 | 0.464789 | 0.225352 | 0.169014 | 0.140845 |
|     | 1 | 0.605263 | 0.434211 | 0.289474 | 0.184211 | 0.078947 |
|     | 1 | 0.507463 | 0.522388 | 0.298507 | 0.19403  | 0.089552 |
|     | 1 | 0.499057 | 0.396838 | 0.278245 | 0.216689 | 0.13552  |
|     | 0 | 0.092792 | 0.070528 | 0.036096 | 0.044154 | 0.035091 |
|     | 1 | 0.464539 | 0.358359 | 0.281812 | 0.233825 | 0.151723 |
|     | 0 | 0.095816 | 0.058329 | 0.039727 | 0.047122 | 0.024689 |

|        |   |          |          |          |          |          |
|--------|---|----------|----------|----------|----------|----------|
| T24-GR |   |          |          |          |          |          |
|        | 1 | 0.907895 | 0.881579 | 0.723684 | 0.644737 | 0.526316 |
|        | 1 | 0.906667 | 0.813333 | 0.76     | 0.626667 | 0.546667 |
|        | 1 | 0.897436 | 0.807692 | 0.820513 | 0.589744 | 0.564103 |
|        | 1 | 0.943953 | 0.884956 | 0.823009 | 0.79646  | 0.725664 |
|        | 1 | 0.930029 | 0.860058 | 0.816327 | 0.728863 | 0.676385 |
|        | 1 | 0.973134 | 0.862687 | 0.844776 | 0.764179 | 0.722388 |
|        | 1 | 0.960526 | 0.776316 | 0.723684 | 0.644737 | 0.539474 |
|        | 1 | 0.946667 | 0.813333 | 0.76     | 0.626667 | 0.573333 |
|        | 1 | 0.897436 | 0.807692 | 0.692308 | 0.589744 | 0.564103 |
|        | 1 | 0.929305 | 0.834183 | 0.773811 | 0.667977 | 0.60427  |
|        | 0 | 0.026697 | 0.036405 | 0.051093 | 0.07165  | 0.075767 |

*Fig 1*

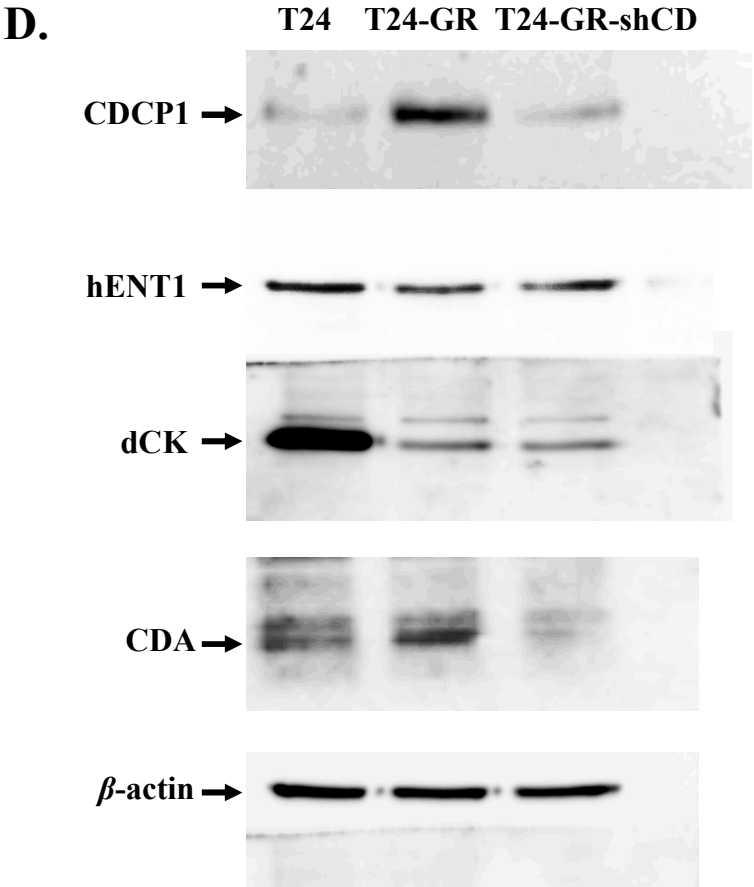

Supplement: S1 Fig — (PDF) [file pone.0331289.s001.pdf]

**Fig 2**

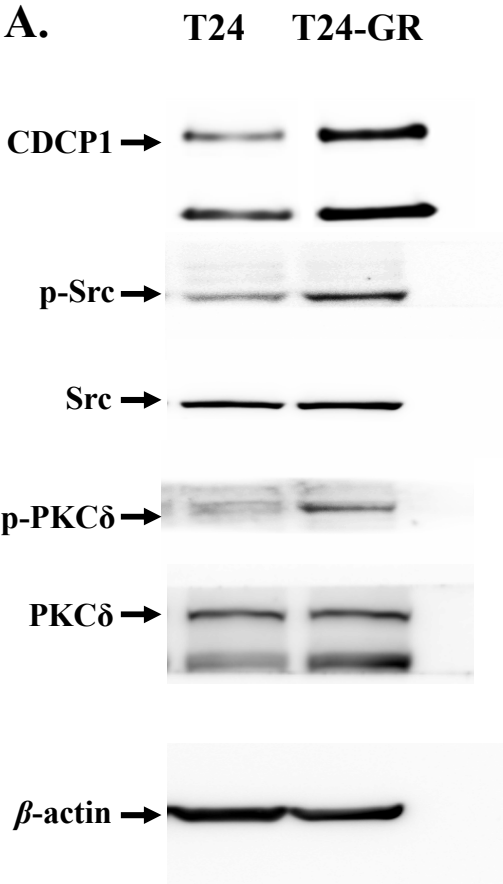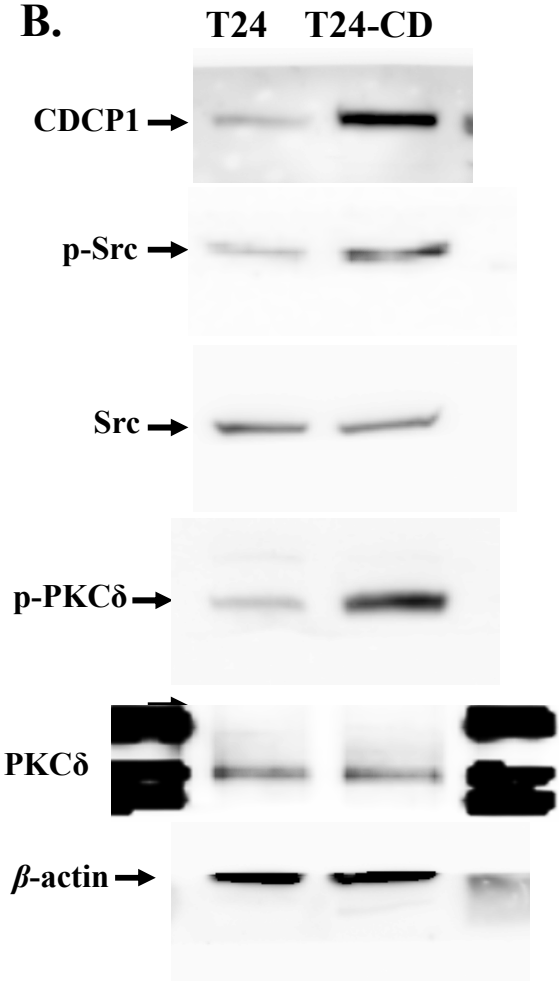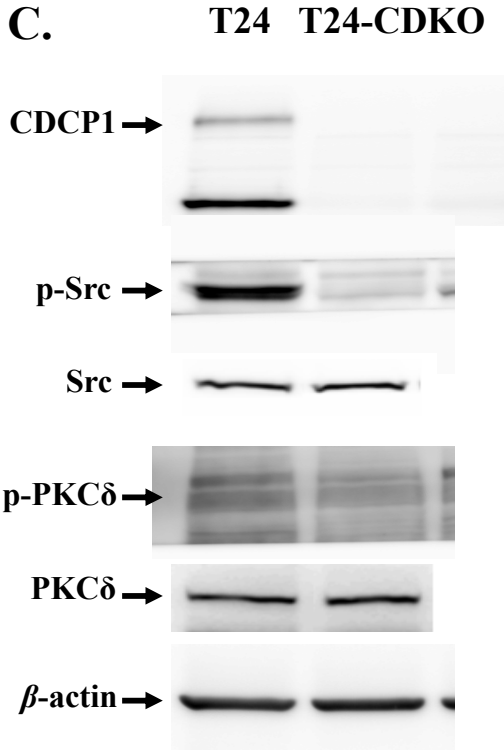

Supplement: S2 Fig — (PDF) [file pone.0331289.s002.pdf]

**Fig 3**

**C. T24-CD**

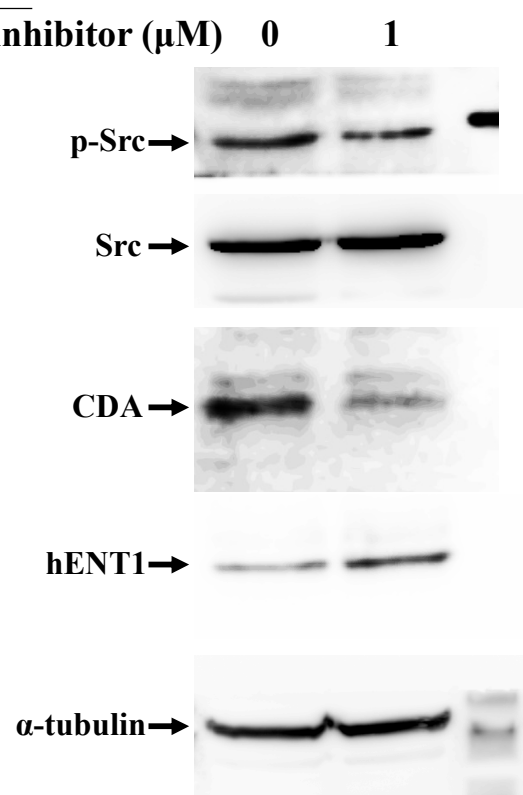

**D. T24-CD**

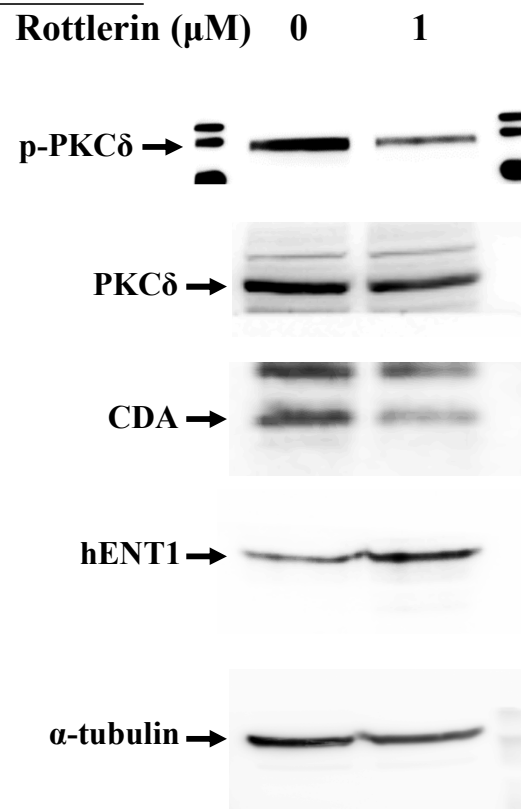

Supplement: S3 Fig — (PDF) [file pone.0331289.s003.pdf]

**Fig 4**  
**D.**

Caspase3

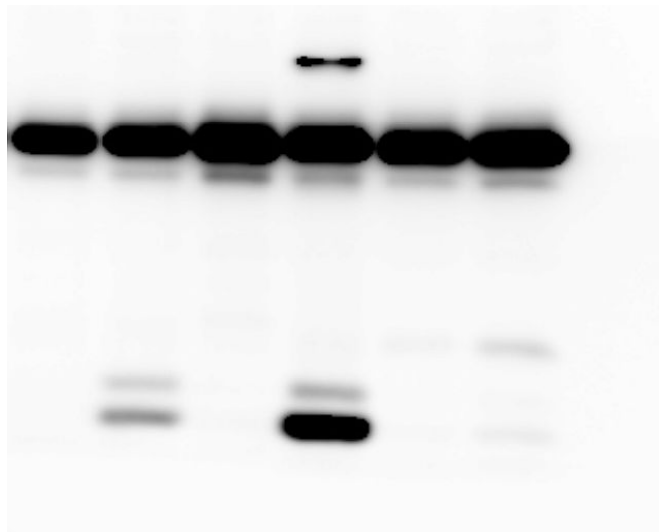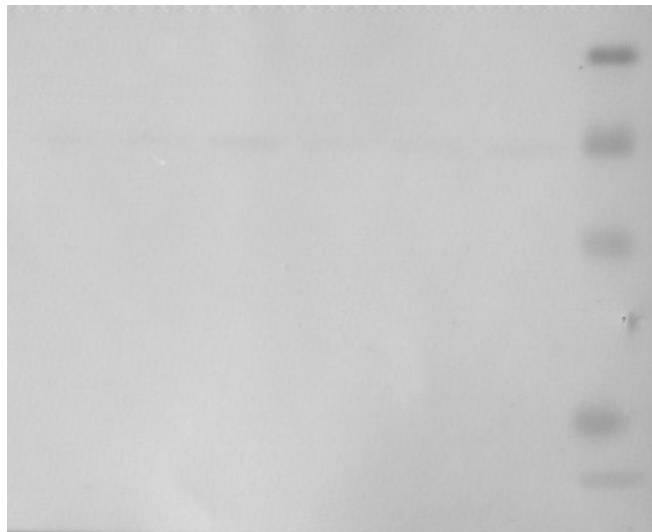

cl-PARP

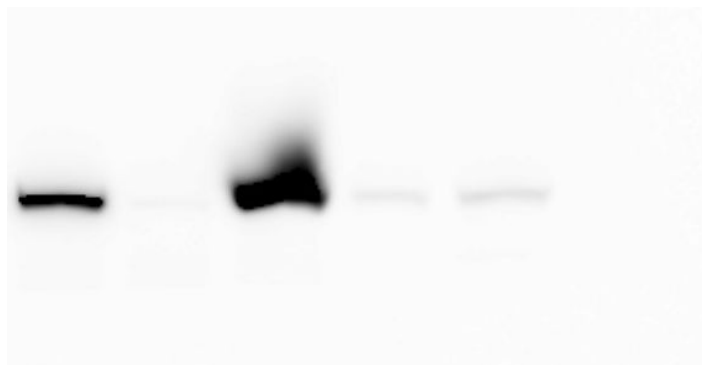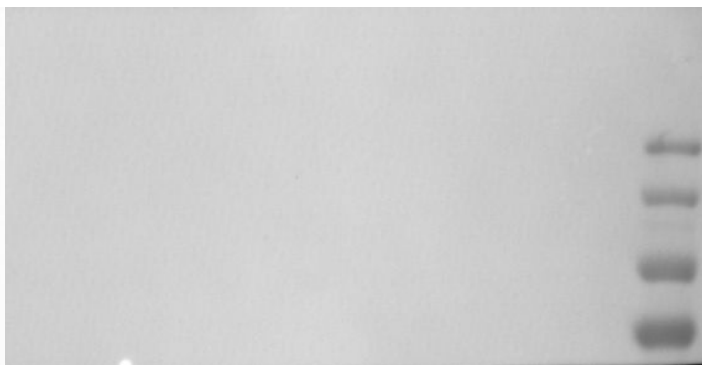

**Fig 4**

**D.**

B-actin

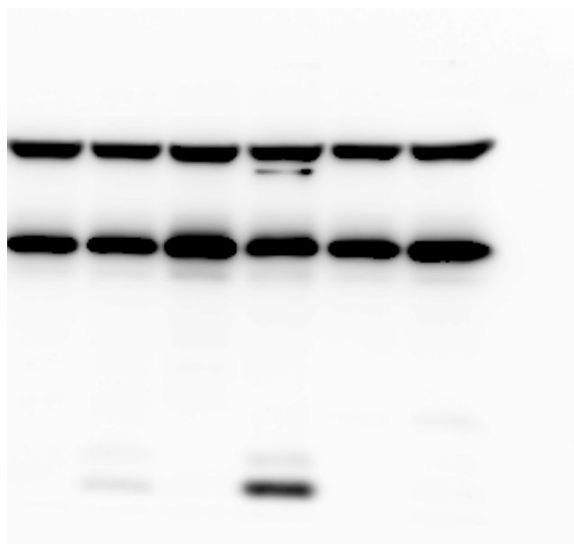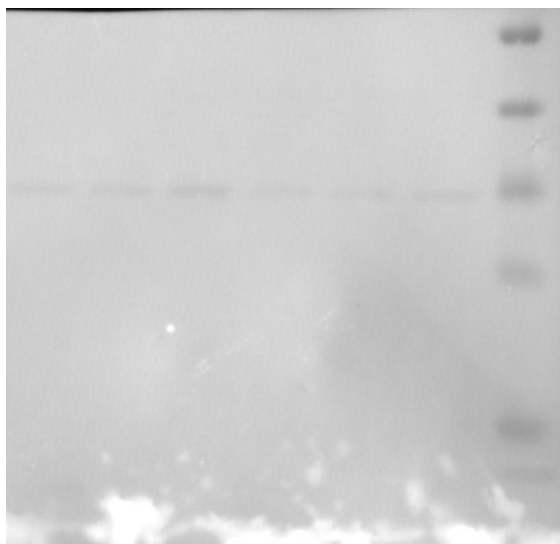

B-actin

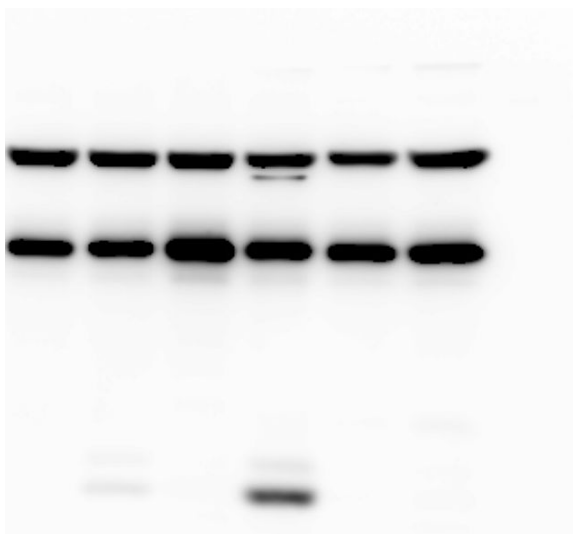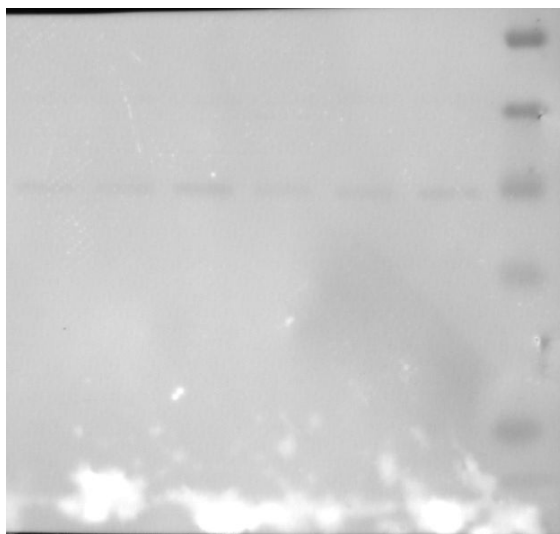

**Fig 4**

**G. T24-CDKO**

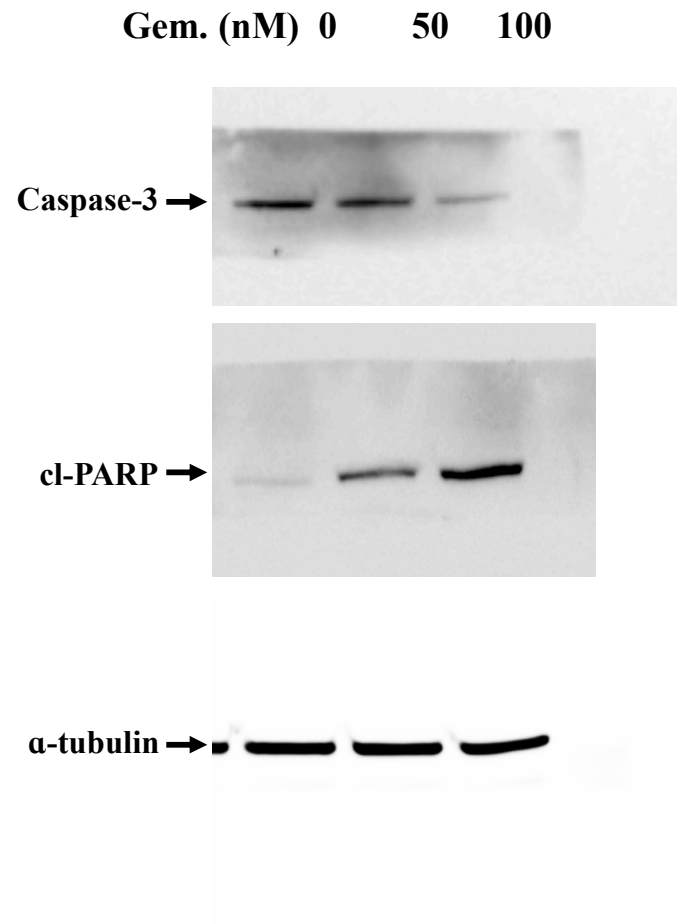

Supplement: S4 Fig — (PDF) [file pone.0331289.s004.pdf]
